# Core outcome set for uncomplicated acute appendicitis in children and young people

**DOI:** 10.1002/bjs.11508

**Published:** 2020-03-17

**Authors:** F. C. Sherratt, B. S. R. Allin, J. J. Kirkham, E. Walker, B. Young, W. Wood, L. Beasant, Appendicitis Core Outcome Set Study Group, D. Rex, K. Kalka, S. Marven, J. Rae, S. Sotirios, S. Braungart, O. Gee, C. Skerritt, B. Lakshminarayanan, R. Lisseter, R. Brampton, L. Luedekke, H. Corbett, S. Eaton, N. J. Hall

**Affiliations:** ^1^ Institute of Population Health Sciences University of Liverpool Liverpool UK; ^2^ National Perinatal Epidemiology Unit University of Oxford Oxford UK; ^3^ Centre for Biostatistics, Manchester Academic Health Science Centre University of Manchester Manchester UK; ^4^ Centre for Outcomes and Experience Research in Children's Health Illness and Disability (ORCHID), Great Ormond Street Hospital for Children NHS Foundation Trust London UK; ^5^ Developmental Biology and Cancer Programme University College London Great Ormond Street Institute of Child Health London UK; ^6^ National Institute for Health Research (NIHR) Research Design Service South Central University of Southampton Southampton UK; ^7^ University Surgery Unit, Faculty of Medicine University of Southampton Southampton UK; ^8^ Bristol Medical School University of Bristol Bristol UK

## Abstract

**Background:**

Research studies to inform clinical practice and policy in children and young people with appendicitis are hampered by inconsistent selection and reporting of outcomes. The aim of this study was to develop a core outcome set for reporting all studies of uncomplicated acute appendicitis in children and young people.

**Methods:**

Systematic literature reviews, qualitative interviews with parents and patients treated for uncomplicated acute appendicitis, and a Study‐Specific Advisory Group informed a long list of outcomes. Outcomes were then prioritized by stakeholders based in the UK (patients, parents, and paediatric and general surgeons) in an online three‐round Delphi consensus process, followed by face‐to‐face consensus meetings.

**Results:**

A long list of 40 items was scored by 147 key stakeholders in the first Delphi round, of whom 90 completed the two subsequent Delphi rounds. The final core outcome set comprises 14 outcomes: intra‐abdominal abscess, reoperation (including interventional radiology procedure), readmission to hospital, bowel obstruction, wound infection, antibiotic failure, wound complication, negative appendicectomy, recurrent appendicitis, death, patient stress/psychological distress, length of hospital stay, time away from full activity and child's quality of life.

**Conclusion:**

A core outcome set comprising 14 outcomes across five key domains has been developed for reporting studies in children and young people with uncomplicated acute appendicitis. Further work is required to determine how and when to measure these outcomes.

## Graphical abstract

Acute appendicitis in children and young people has a variety of outcomes reported. A core outcome set has been developed with paediatric and general surgeons, parents and patients. The core outcome set comprises 14 outcomes and should allow uniform reporting of outcomes that are important for key stakeholder groups.

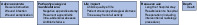

Fourteen key outcomes across five domains

## Abstract (es)

**Antecedentes:**

Los estudios de investigación que sirvan de base para la práctica clínica y la política en niños y adultos jóvenes con apendicitis se ven obstaculizados por inconsistencias en la selección y descripción de los resultados. El objetivo de este estudio fue desarrollar un conjunto central de resultados para todos los estudios de apendicitis aguda no complicada en niños y adultos jóvenes.

**Métodos:**

Para establecer una lista de resultados se efectuaron revisiones sistemáticas de la literatura, entrevistas cualitativas con padres y pacientes tratados por apendicitis aguda no complicada, y consulta con un Grupo de Asesoramiento Específico para el Estudio. Seguidamente, los resultados se priorizaron de acuerdo con los intereses de las partes interesadas (pacientes, padres, y cirujanos pediátricos y generales) en el Reino Unido a través de un proceso de consenso Delphi de tres rondas en Internet, seguido de reuniones personales de consenso.

**Resultados:**

Un total de 147 participantes puntuaron una larga lista de 40 ítems en la primera ronda Delphi, de los cuales 90 completaron las dos rondas Delphi subsiguientes. El conjunto final incluye 14 resultados: absceso intra‐abdominal, reoperación (incluyendo procedimientos radiológicos intervencionistas), reingreso, obstrucción intestinal, infección de herida, otras complicaciones de la herida, fracaso del tratamiento con antibióticos, apendicectomía blanca, apendicitis recidivante, muerte, estrés del paciente/sufrimiento psicológico, duración de la estancia hospitalaria, tiempo alejado de todas sus actividades y calidad de vida.

**Conclusión:**

Se ha desarrollado un conjunto central de resultados que incluye 14 resultados en cinco dominios clave para la descripción de estudios en niños y adultos jóvenes con apendicitis aguda no complicada. Se requieren más trabajos para determinar cómo y cuándo conviene medir estos resultados.

## Introduction

Acute appendicitis is the most common surgical emergency in children and young people^1^. A systematic review^2^ of outcomes reported in RCTs and meta‐analyses of acute appendicitis in children revealed wide heterogeneity in outcome selection. Such differences make it challenging, sometimes impossible, to synthesize study results and conduct meta‐analyses. Standardizing outcomes by developing and implementing a core outcome set facilitates data synthesis, and ensures that outcomes of importance to relevant stakeholders are included. This study aimed to develop a core outcome set for future research on operative and non‐operative treatments for uncomplicated acute appendicitis in children and young people (aged less than 18 years).

## Methods

The study adopted established consensus methods^3^, involving three phases: phase 1, development of a long list of outcomes using systematic reviews and qualitative interviews with patients and parents; phase 2, a three‐round online Delphi survey with key stakeholders (patients, parents, and paediatric and general surgeons); and phase 3, two consensus meetings. The recommended core outcome set development standards were met when planning, conducting and reporting the project^4^, ^5^. The study was part of the CONservative TReatment of Appendicitis in Children – a randomized controlled Trial (CONTRACT) feasibility study^6^ which received research ethical approval (South Central – Hampshire A Research Ethics Committee, 16/SC/0596).

A Study‐Specific Advisory Group (SSAG) comprising children, young people and parents, which was assembled for the CONTRACT feasibility study^7^, informed the development of study materials and a participant video^8^. Some members also attended the patient and parent consensus meeting to facilitate discussion. The study protocol has been published elsewhere^9^ and was registered with the COMET (Core Outcome Measures in Effectiveness Trials) Initiative in May 2017^10^.

### Scope of the core outcome set

The core outcome set is intended for use in future research (including clinical trials) that evaluates the overall success of operative or non‐operative (antibiotics) treatment in children and young people aged less than 18 years with uncomplicated acute appendicitis.

### Phase 1: developing a long list of outcomes

A long list of outcomes was developed from two sources: first, from recent systematic literature reviews^2^, ^11^ to identify previously reported outcomes in trials examining the treatment of uncomplicated acute appendicitis in children and young people; and, second, from the CONTRACT communication study embedded within the CONTRACT feasibility study. This involved semistructured qualitative interviews with patients and parents who participated in the CONTRACT feasibility study^7^ to explore which outcomes were important to them. Researchers subsequently mapped outcomes identified from the qualitative study to outcomes identified from the literature reviews to determine whether there were any additional outcomes. This long list was then refined to avoid duplication. Members of the SSAG were presented with draft text, including outcome names and descriptions, and instructions on outcome scoring, for the Delphi survey website. The group was asked to annotate and discuss the text and design on the Delphi website to improve clarity and comprehensibility. Additional outcomes suggested by the SSAG were also considered for inclusion. The group's feedback was used to revise the Delphi website before invitations were sent to potential participants.

### Phase 2: three‐round online Delphi survey

A three‐round online Delphi survey was used to develop the core outcome set.

#### *Stakeholder identification*

Stakeholders were separated into three panels: patients; parents; and paediatric or general surgeons who treat children with appendicitis. Patients (aged 12–18 years) and parents of patients (aged 5–18 years), who had either received treatment or whose children had received treatment for uncomplicated acute appendicitis in the preceding 24 months, were invited to participate at seven specialist children's hospitals, across five geographical regions in England.

Paediatric surgeons in the UK who treat children with uncomplicated acute appendicitis were invited to participate via the membership list of the British Association of Paediatric Surgeons (including consultants and trainees), and through professional contacts of the investigators. General surgeons who regularly treat children with uncomplicated acute appendicitis were invited to participate via the Association of Surgeons of Great Britain and Ireland, existing professional contacts and regional surgical networks in the UK.

Stakeholders from outside the UK were not invited as this would have posed logistical and resource challenges given that the intention was to include patients, parents and surgeons^3^.

#### *Stakeholder registration*

Stakeholders received an invitation letter and information sheet with a link to a study website, allowing them to access further information, view the study video and register. Registration was open for approximately 10 weeks until the desired number of stakeholders had registered (minimum 10 per panel). The aim was to have 75–100 stakeholders in the Delphi first round with equal numbers in each stakeholder panel^12^.

#### *Delphi surveys*

An online three‐round Delphi survey was carried out in parallel across all stakeholder panels. Software developed by the National Perinatal Epidemiology Unit (University of Oxford, Oxford, UK), which had been used successfully to develop two paediatric surgical core outcome sets^13^, ^14^, was hosted on a secure server.

Stakeholders were presented with the long list of outcomes arranged into themes. In all three Delphi rounds, stakeholders were asked to score each outcome using the GRADE (Grading of Recommendations, Assessment Development and Evaluations) scale, which is commonly used in the development of core outcome sets. The scale allows stakeholders to score the importance of each outcome from 1 to 9 as follows: 1–3, not important; 4–6, important but not critical; and 7–9, critical for inclusion in a core outcome set^15^. Stakeholders could propose other outcomes at the end of the round 1 survey. These were reviewed and mapped to the existing long list, if appropriate. New outcomes were added to the long list in round 2 if: members of the study team were not in agreement that the outcome could be mapped to one on the existing long list; they were defined as an outcome^3^; and were proposed by at least two stakeholders.

All stakeholders who completed each round were invited to participate in the subsequent round. Participants were provided with feedback from their own stakeholder panel in round 2, and from all stakeholder panels in round 3, in order to build consensus between stakeholder panels and then across panels^14^.

Scores for each outcome were analysed for each stakeholder panel and descriptive statistics generated at the end of each round. In accordance with the GRADE scoring system, the consensus status of each outcome was defined at the end of each round. The status was ‘consensus in’ when at least 70 per cent of stakeholders rated the outcome 7–9, and less than 15 per cent rated it as 1–3; ‘consensus out’ when at least 70 per cent of stakeholders rated the outcome 1–3 and less than 15 per cent rated it 7–9; and ‘no consensus’ if the outcome did not meet the criteria for either ‘consensus in’ or ‘consensus out’.

In round 2, all stakeholders were asked to rescore each outcome, taking into account how others in their stakeholder panel had scored the outcome. Stakeholders were also asked to score any new outcomes.

In round 3, attrition was noted among the patient panel. As the aim was to ensure that the final core outcome set represented the views of children and young people, in addition to parents and surgeons, all patients who completed round 1 were invited to participate in round 3. All stakeholders were again asked to rescore each outcome, taking into account how others in their panel and stakeholders in the other two panels had scored the outcome in round 2.

### Phase 3: consensus meetings

The study team e‐mailed surgeons and parents who completed all three rounds of the Delphi survey, together with all patients who had registered for the study (to increase patient representation), to invite them to attend a consensus meeting in Birmingham, UK, in June 2018. However, far fewer stakeholders were able to attend than anticipated, so the study team agreed to postpone the consensus meeting. Following consultation with stakeholders, the team held two separate consensus meetings. Although this deviated from the study protocol, other core outcome set developers have used this approach, and have suggested that holding separate meetings for patients and health professionals may help avoid the risk that meetings are dominated by health professionals^16^. The surgeon consensus meeting was held in September 2018 in London, and the parent and patient consensus meeting in September 2018 in Birmingham.

All attendees were sent a consensus meeting booklet describing the study aims, purpose of the consensus meetings, and an overview of the Delphi results. A chairperson was selected for each meeting with expertise in core outcome set development to promote and potentially mediate discussion. The Patient and Public Involvement Lead for the CONTRACT feasibility study and two families from the SSAG also attended the parent and patient consensus meeting to help facilitate discussion, but did not vote. The meetings included an overview of the study so far, instructions for the meeting, discussion of outcomes and anonymous voting using TurningPoint electronic software (Turning Technologies, Youngstown, Ohio, USA).

Owing to attrition (particularly among patients) and potential response bias, the study team prioritized which outcomes to discuss and rescore in the meetings, rather than rescoring all outcomes as proposed previously^9^. Brief discussion took place for outcomes where at least 70 per cent of stakeholders across all panels rated the outcome 7–9. After discussion, the Chair asked stakeholders whether they felt that any of these outcomes should be excluded from the final core outcome set, and outcomes were rescored only if stakeholders voiced a preference to revote. Again, brief discussion took place for outcomes where less than 50 per cent of stakeholders across all stakeholder panels rated the outcome 7–9 at the end of the third Delphi round, with an option to rescore if stakeholders voiced a preference to revote. All other outcomes (those scored 7–9 by 50–69 per cent of stakeholders) were presented, discussed and rescored, unless stakeholders felt strongly that, following discussion, rescoring was unnecessary. After discussion and rescoring, outcomes reaching ‘consensus in’ at either meeting were included in the final core outcome set, in line with previous studies with a similar design^16^, ^17^. All other outcomes were excluded.

### Data analysis

Descriptive statistics were used to calculate the scores for each outcome, which were analysed in total and for each stakeholder panel, at each round. The data analysis process from round 1 was repeated for rounds 2 and 3. The study team included partial respondents in the analysis, and examined graphically whether attrition had an effect on outcome scores, as recommended in the COMET Handbook^3^, and using a multilevel modelling approach (level 1: outcome; level 2: participant; level 3: stakeholder panel) employing MLwiN version 3.01 (Centre for Multilevel Modelling, University of Bristol, Bristol, UK). Results are shown as estimates with standard deviations.

## Results

### Phase 1: developing a long list of outcomes

The qualitative team identified eight outcomes of importance to families, and all of these mapped to outcomes already identified from the reviews (*Table* *S1*, supporting information). Thus no new outcomes were revealed and a long list of 40 outcomes was generated (*Table* *S2*, supporting information) and categorized into themes: outcomes during an operation (if performed); outcomes that may occur after treatment; duration of recovery; additional (unplanned) procedures during the first hospital admission; outcomes related to pain; other complications; outcomes reported by patients; cost of treatment and resources used; and other outcomes.

### Phase 2: three‐round online Delphi surveys

Between October and December 2017, 818 parents and patients from seven National Health Service sites in England were invited to participate in the study. It was not possible precisely to measure the number of paediatric and general surgeons who were invited to participate.

**Figure 1:**
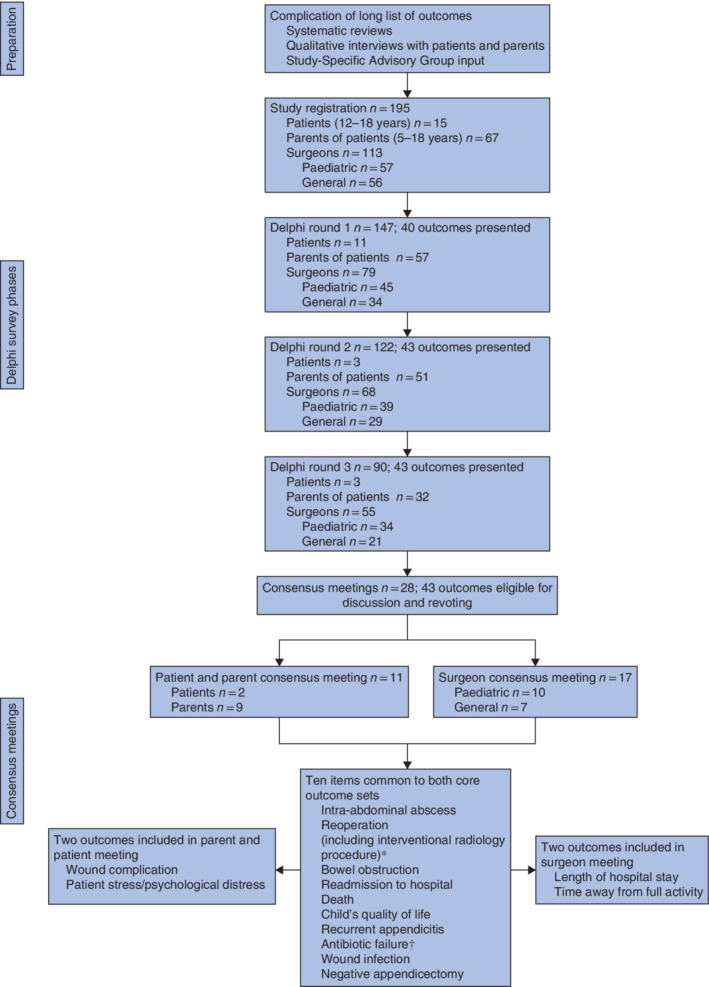
Study flow chart *Reoperation was redefined to include interventional radiology procedure. †It is necessary to report antibiotic failure only in studies of non‐operative treatment.

Overall, 195 stakeholders were registered (15 patients, 67 parents, 57 paediatric surgeons and 56 general surgeons) (*Fig*.  1). The median ages of patients who registered and completed rounds 1, 2 and 3 were 12·5 (range 11–18), 13·5 (11–18), 12 (12–14) and 14 years respectively. The median age of patients of parents who registered and completed rounds 1– 3 was 10 (3–18) years throughout.

Twenty‐six stakeholders in round 1 proposed 35 additional outcomes (*Table* *S3*, supporting information), of which three were agreed to be new and included in subsequent rounds: psychological distress, negative appendicectomy and time to normal diet.

None of the outcomes were voted ‘consensus out’ in any of the Delphi rounds. Outcome scores across each round are summarized in *Table* 1, and outcome scores for each stakeholder panel by Delphi round are shown in *Tables* *S4–S6* (supporting information). Attrition analysis indicated no systematic significant difference in responders and non‐responders between rounds 1 and 2, and rounds 1 and 3. On average, round 1 scores were 6·1(s.d. 0·2) for those who completed round 1 only, and were increased by 0·1(0·2) among those who also completed round 2 (*P* = 0·609); round 1 scores were increased by 0·1(0·3) among those who also completed round 3 (*P* = 0·603). Median scores for individual items, and score frequency distributions were also similar between responders and non‐responders (*Tables* *S7* and *S8*, *Figs* *S1* and *S2*, supporting information).

**Table 1:** Summary of stakeholder outcome ratings across Delphi rounds and final consensus on outcomes at consensus meetings

	Delphi rounds			Consensus meetings	
	No. of stakeholders who voted outcome very important (score 7–9)			Final core outcome set decision	
Outcome	Round 1 (*n* = 147)	Round 2 (*n* = 122)	Round 3 (*n* = 90)	Surgeon meeting (*n* = 17)	Parent and patient meeting (*n* = 11)
Operating time	48 (34·3)†	39 (32·5)†	23 (26)†	Excluded	Excluded
Conversion to open operation	55 (41·0)†	56 (47·5)†	34 (39)†	Excluded	Excluded
Blood loss	85 (63·4)†	93 (78·8)*	67 (76)*	Excluded	Excluded
Wound infection	88 (60·7)†	76 (62·8)†	58 (65)†	Included	Included
Intra‐abdominal abscess	124 (85·5)*	115 (96·6)*	84 (95)*	Included	Included
Wound complication	91 (63·6)†	77 (63·6)†	57 (64)†	Excluded	Included
Fever after treatment	68 (46·9)†	59 (48·8)†	36 (41)†	Excluded	Excluded
Blood markers of inflammation	57 (41·9)†	54 (45·8)†	29 (33)†	Excluded	Excluded
Other infectious complication	59 (42·8)†	53 (44·9)†	29 (33)†	Excluded	Excluded
Duration of antibiotics	47 (33·1)†	35 (29·4)†	14 (16)†	Excluded	Excluded
Recovery of bowel function	51 (35·7)†	43 (35·8)†	22 (25)†	Excluded	Excluded
Time to ambulation	60 (42·9)†	49 (41·2)†	27 (30)†	Excluded	Excluded
Length of hospital stay	70 (47·9)†	54 (45·0)†	48 (54)†	Included	Excluded
Duration of drainage	49 (40·5)†	53 (47·7)†	38 (46)†	Excluded	Excluded
Unplanned CT	40 (30·5)†	35 (30·2)†	25 (29)†	Excluded	Excluded
Any unplanned imaging	32 (24·1)†	25 (21·4)†	15 (18)†	Excluded	Excluded
Interventional radiology procedure	53 (44·5)†	73 (64·6)†	59 (69)*	Excluded‡	Excluded
Unplanned central venous catheter	56 (46·3)†	73 (64·0)†	64 (74)*	Excluded	Excluded
Reoperation	97 (74·6)*	109 (93·2)*	81 (94)*	Included‡	Included
Antibiotic failure§	79 (59·0)†	82 (71·3)*	64 (76)*	Included	Included
Analgesia	45 (31·5)†	43 (36·1)†	25 (29)†	Excluded	Excluded
Pain score	66 (45·5)†	58 (48·7)†	44 (51)†	Excluded	Excluded
Readmission to hospital	101 (71·1)*	102 (86·4)*	75 (88)*	Included	Included
Bowel obstruction	120 (86·3)*	111 (94·1)*	81 (94)*	Included	Included
Recurrent appendicitis	111 (81·0)*	105 (89·7)*	79 (92)*	Included	Included
Major or minor complication	94 (68·1)†	100 (84·7)*	78 (91)*	Excluded	Excluded
Death	124 (87·9)*	112 (94·9)*	81 (95)*	Included	Included
Time away from school	62 (43·1)†	54 (45·8)†	37 (43)†	Excluded	Excluded
Time away from full activity	40 (28·7)†	28 (23·7)†	15 (17)†	Included	Excluded
Parent time off work	34 (23·6)†	32 (27·1)†	16 (19)†	Excluded	Excluded
Wound healing time	41 (28·5)†	37 (31·4)†	23 (27)†	Excluded	Excluded
Child's quality of life	96 (66)†	100 (84·0)*	82 (94)*	Included	Included
Cosmesis	32 (25)†	30 (26·5)†	22 (26)†	Excluded	Excluded
Parental stress	41 (28)†	34 (28·8)†	17 (20)†	Excluded	Excluded
Patient stress	76 (52)†	72 (60·5)†	59 (66)†	Excluded	Included‡
Total cost of treatment	38 (28)†	27 (23·1)†	14 (16)†	Excluded	Excluded
Cost‐effectiveness	57 (42)†	49 (41·5)†	35 (41)†	Excluded	Excluded
Total healthcare visits	37 (27)†	29 (25·0)†	16 (19)†	Excluded	Excluded
Duration of home healthcare	25 (19)†	11 (9·5)†	5 (6)†	Excluded	Excluded
Bacterial peritoneal cultures	36 (29)†	33 (29·2)†	23 (28)†	Excluded	Excluded
Psychological distress¶	–	52 (44·4)†	37 (43)†	Excluded	Included‡
Negative appendicectomy¶	–	41 (37·6)†	29 (35)†	Included	Included
Time to normal diet¶	–	25 (21·0)†	12 (14)†	Excluded	Excluded

Values in parentheses are percentage of stakeholders who scored the item.

* Consensus in;

† no consensus.

‡ Item combined with another item in the final core outcome set.

§ It is necessary to report antibiotic failure only in studies of non‐operative treatment.

¶ Outcome introduced in round 2.

### Phase 3: consensus meetings

Overall, 28 stakeholders participated in the consensus meetings (*Fig*.  1).

#### *Surgeon consensus meeting*

Ten paediatric and seven general surgeons attended. During the meeting, several outcomes warranted extended discussion and/or a second vote. Some were outcomes that were not voted ‘consensus in’ in round 3 of the Delphi survey but which surgeons felt strongly were important to include in the core outcome set: negative appendicectomy, time away from full activity, length of hospital stay, and wound infection. Following discussion and voting, surgeons reached consensus to include these outcomes.

Other outcomes that were discussed had reached ‘consensus in’ in the Delphi survey, but at the meeting surgeons did not reach consensus regarding including these outcomes. Unplanned central venous catheter was felt to be a complication (rather than an outcome), rare in the context of uncomplicated appendicitis and a measure of practice rather than treatment success. Blood loss was felt not specific enough and surgeons proposed that it was not important unless a transfusion was required. The outcome was redefined to blood loss requiring transfusion and a subsequent vote held, but this still did not achieve ‘consensus in’. Surgeons also felt that major or minor complications was too vague and could include too many things for it to be meaningfully included in a core outcome set.

Two other outcomes were discussed by surgeons. It was proposed and agreed that interventional radiology procedure should be combined with reoperation, another outcome that achieved ‘consensus in’, because in a child they would both be procedures under general anaesthesia. Other infectious complication was discussed further with reference to the implications that this complication could have. Surgeons felt that an infectious complication would be deemed as critically important if it resulted in reoperation, but less important if it did not. They therefore agreed that other infectious complication should not be included in the core outcome set, because reoperation had already achieved ‘consensus in’.

In total, surgeons agreed to include 12 outcomes in the core outcome set: intra‐abdominal abscess, reoperation (including interventional radiology procedure), bowel obstruction, readmission to hospital, death, child's quality of life, recurrent appendicitis, antibiotic failure (in studies examining non‐operative treatment), wound infection, length of hospital stay, negative appendicectomy, and time away from full activity.

#### *Patient and parent consensus meeting*

In addition to the seven parents and one patient who had completed the third round of the Delphi survey, three further stakeholders attended. One was a patient (identified via a parent) who matched the study eligibility criteria but had not completed any of the previous Delphi surveys. The other two were parents who wished to accompany their respective partners to the meeting. These additional stakeholders reported completing the Delphi surveys alongside their partner. The study team discussed these cases, and agreed that the additional patient and two parents could attend the meeting to increase patient and parent representation in the core outcome set development.

Again, during the meeting, several outcomes warranted extended discussion and/or a second vote. In round 3 of the Delphi survey, patients and parents did not reach consensus regarding inclusion of psychological distress, and parents were not in consensus about inclusion of negative appendicectomy; however, in the meeting, patients and parents reached consensus to include both outcomes. Patient stress achieved ‘consensus in’, and with one additional vote psychological distress would also have achieved ‘consensus in’. Following discussion, it was agreed to include patient stress/psychological distress as a combined outcome in the final core outcome set.

In Delphi round 3, patients and parents reached consensus to include major or minor complications, pain score and fever after treatment, and parents were in consensus to include blood loss. However, patients and parents did not reach consensus regarding inclusion of any of these outcomes following discussion at the meeting. Pain score and fever after treatment were not felt to be critically important, and participants agreed that the importance of blood loss would depend on the amount of blood lost and its effect. Parents and patients suggested that the important aspect of major or minor complications was the need to capture a major complication but that a minor complication was less important. As key major complications, such as intra‐abdominal abscess and bowel obstruction, were already included there was agreement not to include major or minor complications.

In total, parents and patients agreed to include 12 outcomes in the core outcome set: intra‐abdominal abscess, reoperation (including interventional radiology procedure), bowel obstruction, readmission to hospital, death, child's quality of life, recurrent appendicitis, antibiotic failure (although it was suggested to include this only in studies examining non‐operative treatment), wound infection, wound complication, negative appendicectomy, and patient stress/psychological distress.

#### *Finalizing the core outcome set*

**Figure 2:**
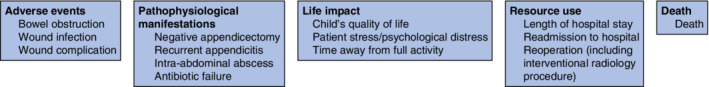
Final core outcomes grouped by OMERACT domains OMERACT, Outcome Measures in Rheumatology.

Combining the surgeon and patient and parent outcome sets resulted in a final core outcome set of 14 outcomes, including antibiotic failure, which needs to be measured and reported only in studies of non‐operative treatment (*Fig*.  1). The outcomes were aligned with OMERACT (Outcome Measures in Rheumatology) Filter 2.0^18^ domains (*Fig*.  2).

## Discussion

The final core outcome set includes 14 outcomes. Further work is necessary, following COSMIN (COnsensus‐based Standards for the selection of health Measurement Instruments) to define and measure the core outcomes^19^, for example selection of the appropriate measure for child's quality of life.

One of the strengths of the study was the inclusion of children, young people and parent stakeholders to ensure that important outcomes were considered and included that might otherwise be overlooked^20^. Although adult patients have been increasingly included in the development of core outcome sets^3^, few studies of core outcome sets have included both children and parents as stakeholders. Of those that have^21^, ^22^, the degree to which children and parents have been able to contribute their views on which outcomes are important at all stages of the consensus process has varied. Engaging these important stakeholders in the development of the present core outcome set and maintaining their engagement was challenging. To an extent, the study team anticipated this; young people and parents from the SSAG were consulted in advance to inform study materials, and methods adjusted to optimize engagement. Despite this, the proportion of young people and parents who registered and engaged in all stages of the study was lower than anticipated. When this was discussed with families, they indicated that, having recovered from appendicitis, patients and parents had moved on with their lives such that appendicitis was often something that had been forgotten. Furthermore, as uncomplicated appendicitis is an acute condition and the extent of involvement of health professionals from various disciplines is arguably more limited than for chronic conditions, the study team did not include an expansive range of health professional stakeholder panels. The focus was primarily on those likely to be most affected (young people and parents) and those who typically determine treatment decisions (surgeons). Previous studies^23^, ^24^, ^25^ have adopted a similar approach, but it is acknowledged that methods may have been strengthened by including a broader range of health professional stakeholder roles.

A potential limitation of this study is that it was conducted in the UK only. Although this strengthens the validity of the core outcome set for future UK‐based research, international ratification or validation of the core outcome set should take place before adoption in other countries. Researchers planning to use this core outcome set in other geographical areas should determine its applicability to their research setting by using critical appraisal tools, such as COS‐STAD (Core Outcome Set – STAndards for Development)^5^. A protocol for an international core outcome set for treatment of uncomplicated appendicitis in children has been published recently^26^.

Having developed a core outcome set for studies in uncomplicated acute appendicitis in children and young people using robust consensus methods, the surgical community should now adopt the core outcome set for future studies. Such adoption will optimize the quality of research in this field, and ensure that the outcomes measured are clinically relevant to patients, parents and surgeons. Future work is needed to agree how best the core outcomes should be measured.

### Collaborators

The following members of the Appendicitis Core Outcome Set Study Group are collaborators in this study: D. Rex, K. Kalka (St George's Hospital, London); S. Marven, J. Rae (Sheffield Children's Hospital, Sheffield); S. Sotirios, S. Braungart (Royal Manchester Children's Hospital, Manchester), O. Gee, C. Skerritt (Birmingham Children's Hospital, Birmingham), B. Lakshminarayanan, R. Lisseter (Leeds General Infirmary, Leeds); R. Brampton, L. Luedekke (Southampton Children's Hospital, Southampton); H. Corbett (Alder Hey Hospital, Liverpool).

## Editor's comments

*BJS* remains committed to improving the quality of clinical studies across surgery. Standardization of outcome reporting is key in the drive to improve the quality of the surgical literature. In that regard, this paper is a step in the right direction. Sherratt *et al*. have published a core outcome set (CoS) for young people with appendicitis. A CoS is an agreed set of outcomes that should be measured and reported, as a minimum, in all clinical trials in specific areas of health or healthcare^1^. The CoS methodology was developed using the COMET (Core Outcome Measures in Effectiveness Trials) Initiative, which included input from patients as well as surgeons. A CoS enables comparison of the important outcomes of interventions, improves the ability to perform meaningful systematic review and meta‐analysis, and provides reassurance that the outcomes being reported are the most important to surgeons and patients alike.

*BJS* anticipates CoS will become available for most common surgical conditions in the coming years. We have already published CoS in breast reconstruction surgery and remain keen to publish others^2^. It will become standard for *BJS* to request that authors publish their outcomes according to CoS that are available.

R. J. Hinchliffe

*Editor, BJS*

**References**

1 COMET Initiative. Core Outcome Measures in Effectiveness Trials. http://www.comet-initiative.org.

2 Potter S, Holcombe C,Ward JA, Blazeby JM; BRAVO Steering Group. Development of a core outcome set for research and audit studies in reconstructive breast surgery. *Br J Surg* 2015; **102**: 1360–1371.

## Supporting information


**Appendix S1.** Supporting information
