# Supplementary material for: Core outcome set for uncomplicated acute appendicitis in children and young people
Source: Br J Surg. 2020 Mar 17;107(8):1013–22. doi: 10.1002/bjs.11508 (PMC7317752; doi:10.1002/bjs.11508)

**BJS11508**

**Core outcome set for uncomplicated acute appendicitis in children and young people**

F. C. Sherratt, B. S. R. Allin, J. J. Kirkham, E. Walker, B. Young, W. Wood, L. Beasant, Appendicitis Core Outcome Set Study Group, S. Eaton and N. J. Hall

**Table S1** **Mapping of outcomes generated from the qualitative interviews with patients and parents to outcomes generated from the systematic reviews**

| **Outcome from qualitative work** | **Mapped outcome from systematic reviews** |
| --- | --- |
| Pain | Pain score or analgesia |
| Loss of appetite | Recovery of bowel function |
| Readmission to hospital | Readmission to hospital |
| Wound healing | Wound infection, wound healing time, or wound complication. |
| Child’s psychological wellbeing | Child’s quality of life |
| Time away from school | Time away from school |
| Time away from physical activity | Time away from full activity |
| Post-treatment fever | Post-treatment fever |

**Table S2** **Long list of outcomes and descriptions presented in round one of the Delphi survey (Explanation is given where the outcome is different from the original 37 mapped outcomes).**

| **Outcome** | **Description** |
| --- | --- |
| Intra-abdominal abscess | A pocket of infected fluid or pus deep inside the tummy that may occur after appendicectomy of treatment with antibiotics and may require another procedure or more treatment with antibiotics. |
| Re-operation *(wording changed from ‘ Need for operation/re-operation)* | Having another operation that was not planned. |
| Bowel obstruction *(wording changed from ‘Adhesive obstruction’)* | A blockage of the intestine that would require treatment in hospital on may require an operation to treat it. |
| Major or minor complication | Any type of complication classified as a minor or major (excluding readmission to hospital, bowel obstruction and recurrent appendicitis). |
| Readmission to hospital | Needing to be readmitted to hospital with a stay at least one night. |
| Total healthcare visits *(wording changed from ‘Healthcare visits’)* | How many times the child visits a healthcare professional after they go home following their initial hospital treatment. |
| Any unplanned imaging *(wording changed from ‘Post-treatment imaging’)* | Having any type of x-ray or ultrasound test (other than CT scan) after treatment. |
| Total cost of treatment *(wording changed from ‘Total charges’)* | The total cost of treatment for the health service. |
| Duration of antibiotics | How long a child is treated with antibiotics for. |
| Duration of home healthcare | How long additional healthcare is needed at home after the child’s initial hospital treatment. |
| Death | Dying (please bear in mind that the risk of dying from appendicitis is very low, but it may still be important to measure this as an outcome in studies). |
| Quality of life *(wording changed from ‘Paediatric quality of life’)* | The child’s quality of life that is measured using a specifically designed questionnaire. |
| Recurrent appendicitis | Getting appendicitis again. |
| Antibiotic failure *(wording simplified from ‘Complication of antibiotics or treatment intervention’ as other complications can be included in ‘major/minor complications’)* | Operation to remove the appendix, due to antibiotic failure. |
| Blood Loss *(from additional 9 unmapped outcomes)* | Blood loss during the operation – losing lots of blood during an operation is unusual but you might think it is important to measure this. |
| Unplanned central venous catheter *(from additional 9 unmapped outcomes)* | Having a central venous catheter or not. A catheter is a fine tube inserted into a large vein to give medicines and usually used when medicines in to a vein are likely to be needed for over a week. |
| Wound infection | An infection at the site of the operation that requires treatment with antibiotics. |
| Wound complication (*wording changed from ‘non-infectious wound complication’)* | A complication with the surgical wound, such as the wound opening up before healing, or bleeding. This may need treatment with medicine or another procedure. |
| Interventional radiology procedure | Whether a child needs an interventional radiology procedure or not. Ain interventional radiology procedure is where an x-ray doctor puts a tube inside the tummy to drain pus, using an x-ray for guidance. |
| Patient stress *(proposed by*  *Study-Specific Advisory Group)* | A measure of stress in the child that would be measured using a specifically designed questionnaire. |
| Hospital length of stay | How long a child has to spend in hospital. |
| Pain score | How severe a child reports that pain is, using a pain score. |
| Fever after treatment | A high temperature after treatment has started. |
| Duration of drainage | If a small tube (called a drain) is used after an operation or to drain pus from the tummy, the length of time this is needed for. This is unusual after treatment of uncomplicated appendicitis but can sometimes be needed. |
| Time away from school | How long a child has to spend away from school or their normal activities. |
| Other infectious complication | Other infection during or after treatment that is not related to the appendix or surgical wound. For example, a urine infection or chest infection. |
| Blood markers of inflammation | Results of blood tests that indicate how well the child is responding to treatment. |
| Analgesia (pain relief) | Number of doses and types of painkiller medicine (analgesia) that are needed. |
| Bacterial peritoneal cultures | Which bacteria are grown from inside a patient’s tummy when fluid is tested. |
| Wound healing time *(from additional 9 unmapped outcomes)* | How long the wound takes to heal after an operation. |
| Cosmesis | Cosmesis is the neatness of a wound and whether the child or parent is happy with how it looks. |
| Time away from full activity | How long a child spends away from full activity, such as sport. |
| Cost effectiveness | Cost effectiveness involved working out how much a treatment costs, while also considering whether the treatment worked or not. |
| Conversion to open operation | If an operation that started out as keyhole (a few small holes) needs to be converted to an open operation (a larger cut). |
| Unplanned CT scan *(from additional 9 unmapped outcomes,*  *Study-Specific Advisory Group*  *suggested it was distinct enough from ‘Any unplanned imaging’ to be a separate outcome)* | Whether a CT (CAT) scan is needed after treatment or not. A CT scan makes more detailed pictures of parts of your body than an x-ray but uses higher dose of x-rays. |
| Time to ambulation (or get out of bed) | How long before the child can walk around or move normally. |
| Operation time | Time taken for the operation including time that the child is asleep for (under general anaesthetic). |
| Recovery of bowel function | How long it takes to be able to eat or pass a stool normally. |
| Parental stress *(from additional 9 unmapped outcomes)* | A measure of stress in the parent/guardian that would be measured using a specifically designed questionnaire. |
| Parent time off work *(added as separate outcome following discussion of updated review, as distinct from parental stress, from ‘parent disability days’ ^1^)* | How long a parent/guardian spends away from work. |

1. Minneci PC, Mahida JB, Lodwick DL, Sulkowski JP, Nacion KM, Cooper JN, Ambeba EJ, Moss RL, Deans KJ. Effectiveness of Patient Choice in Nonoperative vs Surgical Management of Pediatric Uncomplicated Acute Appendicitis. *JAMA Surg* 2016;**151**(5): 408-415.

**Table S3 Proposed additional outcomes following Delphi round one.**

| **Summary of additional outcomes proposed by participants in round one** | **Mapped to existing outcome on long list** | **New outcome created: Yes – plus new outcome name**  **No – plus study team reasoning** |
| --- | --- | --- |
| Signs of illness / acuity on blood gases (e.g. raised lactate) | No | No: Not proposed by at least two participants. |
| If the child continues to be in pain after surgery or suffers any type of abdominal pains continuously. | Pain score; Analgesia (pain relief) | No: Outcome mapped to long list. |
| Catheterisation/ urinary issues. | No | No: Not proposed by at least two participants. |
| Bowel habit - constipation due to bowel obstruction/pain; diarrhoea as a marker of intra-abdominal sepsis. | Recovery of bowel function; Other infectious complication. | No: Outcome mapped to long list. |
| Longer term effects of psychological trauma on patients following emergency surgery. | No | Yes: Psychological distress. |
| Wrong diagnosis/negative appendicitis. | No | Yes: Negative appendicectomy. |
| For operative treatment, negative appendicectomy. | No | Yes: Negative appendicectomy. |
| Nausea and vomiting. | No | No: Not proposed by at least two participants. |
| Time to return of normal diet. | No | Yes: Time to normal diet. |
| A proper diagnosis is a good outcome. | No | No: Not classified as an outcome* |
| More information at local hospitals about the treatment of children with appendicitis. Children's hospital diagnosed very quickly, local hospital were quite unsure and did not seem set up to deal with children who have appendicitis and reluctant to make a diagnosis offering an overnight stay. Children's hospital were much quicker and removed the appendix the same day of admission. | No | No: Not classified as an outcome* |
| When recovering from appendicitis, I felt pressure in my shoulders and ribs from extra gas in my body. This extra gas was put into my body to keep the organs in place, as the Doctors told me. It is also a very uncomfortable pain that I think is important in terms of recovery, as it stopped me from moving around well/comfortably, and so on. | Pain score; Analgesia (pain relief) | No: Outcome mapped to long list. |
| Whether girl's fertility would be affected if appendicitis not treated appropriately - this was my concern. | No | No: Not proposed by at least two participants. |
| Longer term complications including obstruction and chronic pain. | Bowel obstruction; Pain score; Analgesia. | No: Outcome mapped to long list. |
| The medico-legal aspects must be taken into consideration. | No | No: Not proposed by at least two participants. |
| Nutritional status. | No | Yes: Time to normal diet. |
| Multiple operations. | Re-operation | No: Outcome mapped to long list. |
| Time to eat. | No | Yes: Time to normal diet. |
| Do we need to do wash out? What antibiotics? | No | No: Not classified as an outcome* |
| Trauma experience after surgery and the time taken to 'get back to normal'. | No | Yes: Psychological distress. |
| Total parenteral nutrition (TPN) usage. | No | No: Not proposed by at least two participants. |
| Surgeon seniority. | No | No: Not classified as an outcome* |
| Appendix pathway in use. | No | No: Not classified as an outcome* |
| Ultrasound scan. | Any unplanned imaging | No: Outcome mapped to long list. |
| Day case (same day discharge). | Hospital length of stay | No: Outcome mapped to long list. |
| Appendicectomy rates. | No | No: Not classified as an outcome* |
| Psychological trauma of the patient. | No | Yes: Psychological distress. |
| Tests parents can do at home to help diagnose appendicitis. | No | No: Not classified as an outcome* |
| Correct pain relief sent home. | Pain score; Analgesia (pain relief) | No: Outcome mapped to long list. |
| Weight loss during hospital admission. | No | No: Not proposed by at least two participants. |
| Anxiety at induction to anaesthetic. | Patient stress | No: Outcome mapped to long list. |
| Accuracy of pre-operative USS in seeing the appendix and diagnosing appendicitis and if this is used routinely, selectively, or not at all. | No | No: Not classified as an outcome* |
| A key measure is the negative appendicectomy rate which should be less than 5%. | No | Yes: Negative appendicectomy. |
| If other imaging modalities are used like MRI and CT and if so, why. | Any unplanned imaging; Unplanned CT scan | No: Outcome mapped to long list. |

* We adopted the COMET definition of an outcome,^1^ i.e. “An outcome is defined to be a measurement or observation used to capture and assess the effect of treatment such as assessment of side effects (risk) or effectiveness (benefits).”

1. Williamson PR, Altman DG, Bagley H, Barnes KL, Blazeby JM, Brookes ST, Clarke M, Gargon E, Gorst S, Harman N, Kirkham JJ, McNair A, Prinsen CAC, Schmitt J, Terwee CB, Young B. The COMET Handbook: version 1.0. *Trials* 2017;**18**(3): 280.

**Table S4 Consensus matrix for outcomes rated ‘consensus in’ by patients across phases**

| **Outcome** | **Phase one** | **Phase two** | **Phase three** |
| --- | --- | --- | --- |
| Conversion to open operation | ✓ | ✓ |  |
| Blood Loss |  | ✓ |  |
| Wound infection |  | ✓ | ✓ |
| Intra-abdominal abscess | ✓ | ✓ | ✓ |
| Wound complication |  | ✓ |  |
| Fever after treatment |  | ✓ | ✓ |
| Blood markers of inflammation | ✓ | ✓ | ✓ |
| Other infectious complication |  | ✓ | ✓ |
| Duration of drainage | ✓ | ✓ | ✓ |
| Unplanned central venous catheter |  | ✓ | ✓ |
| Re-operation |  | ✓ | ✓ |
| Antibiotic failure | ✓ | ✓ | ✓ |
| Analgesia |  |  | ✓ |
| Pain score |  | ✓ | ✓ |
| Readmission to hospital |  |  | ✓ |
| Bowel obstruction |  | ✓ | ✓ |
| Major or minor complication |  | ✓ | ✓ |
| Death | ✓ | ✓ |  |
| Time away from school |  |  | ✓ |
| Time away from full activity |  |  | ✓ |
| Wound healing time |  |  | ✓ |
| Child’s quality of life | ✓ | ✓ |  |
| Cosmesis |  | ✓ | ✓ |
| Patient stress |  | ✓ |  |
| Cost effectiveness |  | ✓ |  |
| Bacterial peritoneal cultures |  | ✓ | ✓ |
| Negative appendicectomy |  |  | ✓ |

✓ Indicates where the definition of ‘consensus in’ was achieved. Grey outcomes highlighted those that were voted ‘consensus in’ by patients across phases

**Table S5 Consensus matrix for outcomes rated ‘consensus in’ by parents across phases**

| **Outcome** | **Phase one** | **Phase two** | **Phase three** |
| --- | --- | --- | --- |
| Conversion to open operation |  | ✓ |  |
| Blood Loss | ✓ | ✓ | ✓ |
| Wound infection | ✓ |  | ✓ |
| Intra-abdominal abscess | ✓ | ✓ | ✓ |
| Wound complication | ✓ | ✓ | ✓ |
| Fever after treatment | ✓ | ✓ | ✓ |
| Blood markers of inflammation |  | ✓ |  |
| Other infectious complication |  | ✓ |  |
| Duration of drainage |  | ✓ |  |
| Re-operation | ✓ | ✓ | ✓ |
| Antibiotic failure | ✓ | ✓ | ✓ |
| Pain score |  | ✓ | ✓ |
| Readmission to hospital | ✓ | ✓ | ✓ |
| Bowel obstruction |  | ✓ | ✓ |
| Recurrent appendicitis | ✓ | ✓ | ✓ |
| Major or minor complication |  | ✓ | ✓ |
| Death | ✓ | ✓ | ✓ |
| Child’s quality of life | ✓ | ✓ | ✓ |
| Patient stress | ✓ | ✓ | ✓ |

✓ Indicates where the definition of ‘consensus in’ was achieved. Grey outcomes highlighted those that were voted ‘consensus in’ by parents across phases

**Table S6** **Consensus matrix for outcomes rated ‘consensus in’ by surgeons across phases**

| **Outcome** | **Phase one** | **Phase two** | **Phase three** |
| --- | --- | --- | --- |
| Blood Loss |  | ✓ | ✓ |
| Intra-abdominal abscess | ✓ | ✓ | ✓ |
| Interventional radiology procedure |  | ✓ | ✓ |
| Unplanned central venous catheter |  |  | ✓ |
| Re-operation | ✓ | ✓ | ✓ |
| Readmission to hospital | ✓ | ✓ | ✓ |
| Bowel obstruction | ✓ | ✓ | ✓ |
| Recurrent appendicitis | ✓ | ✓ | ✓ |
| Major or minor complication | ✓ | ✓ | ✓ |
| Death | ✓ | ✓ | ✓ |
| Child’s quality of life |  | ✓ | ✓ |

✓ Indicates where the definition of ‘consensus in’ was achieved. Grey outcomes highlighted those that were voted ‘consensus in’ by surgeons across all three phases

**Table S7 Phase one scores compared between participants who completed phases one and two, and those who completed phase one only.**

| **Outcome** | **Phase 1 scores for completed phases 1 and 2,**  **Median (range)** | **Phase 1 scores for completed phase 1 only,**  **Median (range)** |
| --- | --- | --- |
| Operation time | 6 (1-9) | 5 (1-9) |
| Conversion to open operation | 6 (1-9) | 5 (2-9) |
| Blood Loss | 7.5 (1-9) | 7 (1-9) |
| Wound infection | 7 (1-9) | 6 (1-9) |
| Intra-abdominal abscess | 8 (3-9) | 8 (2-9) |
| Wound complication | 7 (3-9) | 7 (1-9) |
| Fever after treatment | 6 (1-9) | 7 (1-9) |
| Blood markers of inflammation | 6 (1-9) | 7 (1-9) |
| Other infectious complication | 6 (1-9) | 6 (1-9) |
| Duration of antibiotics | 6 (1-9) | 6 (1-9) |
| Recovery of bowel function | 6 (1-9) | 5 (1-9) |
| Time to ambulation | 6 (1-9) | 6 (3-9) |
| Hospital length of stay | 6 (1-9) | 7 (3-9) |
| Duration of drainage | 6 (1-9) | 6 (1-9) |
| Unplanned CT scan | 5 (1-9) | 5.5 (1-9) |
| Any unplanned imaging | 5 (1-9) | 5 (1-9) |
| Interventional radiology procedure | 6 (1-9) | 5 (1-9) |
| Unplanned central venous catheter | 7 (1-9) | 5 (1-9) |
| Re-operation | 8 (1-9) | 8 (1-9) |
| Antibiotic failure | 7 (1-9) | 8 (3-9) |
| Analgesia | 6 (1-9) | 5 (1-9) |
| Pain score | 6 (2-9) | 6 (1-9) |
| Readmission to hospital | 7 (4-9) | 7 (3-9) |
| Bowel obstruction | 8 (3-9) | 7 (3-9) |
| Recurrent appendicitis | 8 (3-9) | 8 (1-9) |
| Major or minor complication | 8 (3-9) | 8 (3-9) |
| Death | 9 (1-9) | 9 (3-9) |
| Time away from school | 6 (1-9) | 6 (1-9) |
| Time away from full activity | 6 (1-9) | 6 (2-9) |
| Parent time off work | 5 (1-9) | 6 (1-9) |
| Wound healing time | 6 (1-9) | 6 (3-9) |
| Child’s quality of life | 7 (1-9) | 7 (3-9) |
| Cosmesis | 5 (1-9) | 5 (1-9) |
| Parental stress | 5 (1-9) | 5 (1-9) |
| Patient stress | 7 (1-9) | 6 (1-9) |
| Total cost of treatment | 5 (1-9) | 5 (1-9) |
| Cost effectiveness | 6 (1-9) | 6 (1-9) |
| Total healthcare visits | 5 (1-9) | 5 (1-9) |
| Duration of home healthcare | 5 (1-9) | 6 (1-9) |
| Bacterial peritoneal cultures | 5 (1-9) | 5 (1-9) |

**Fig. S1 Phase one scores between participants who completed phases one and two (green), and those who completed phase one only (blue).**


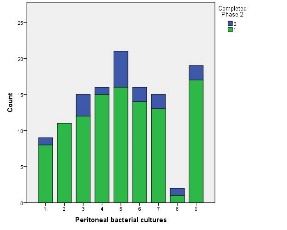

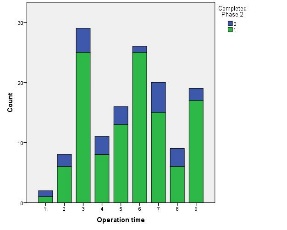

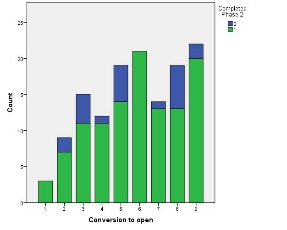

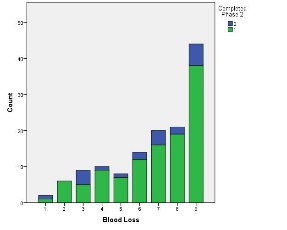

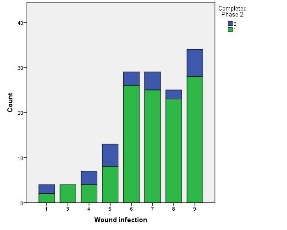

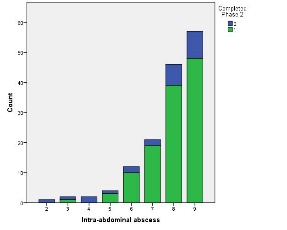

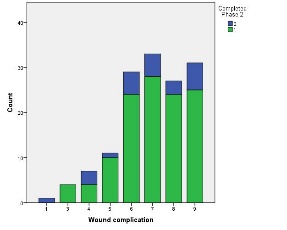

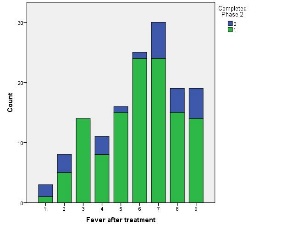

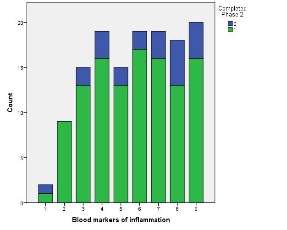

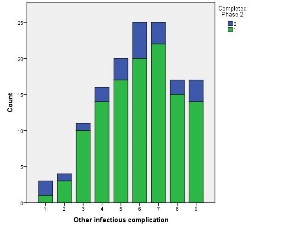

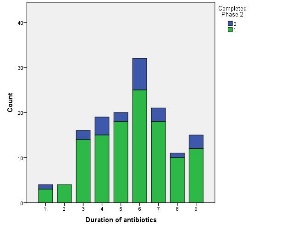

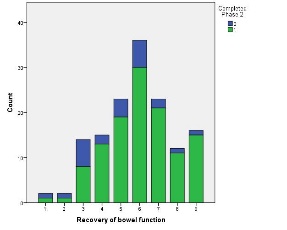

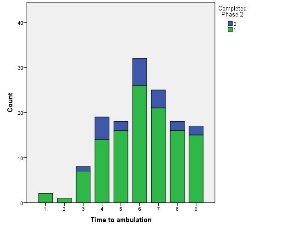

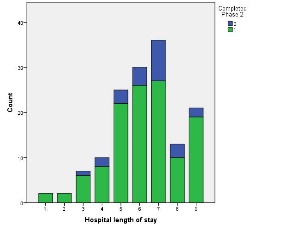

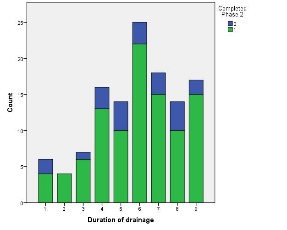

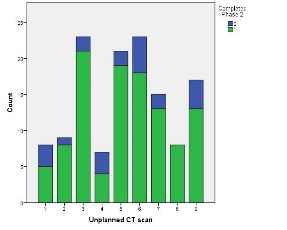

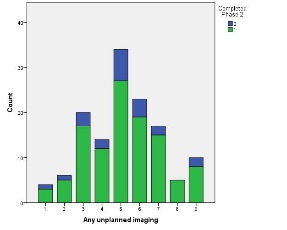

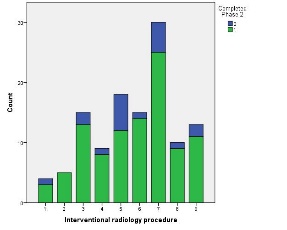

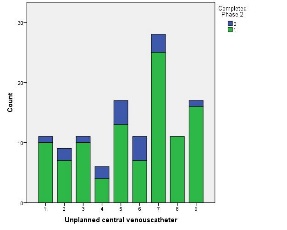

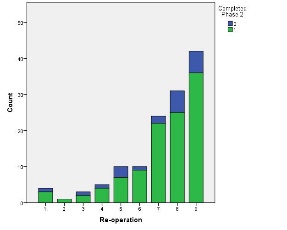

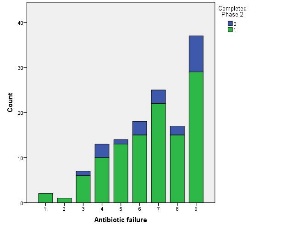

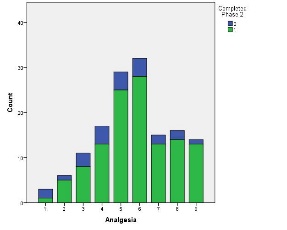

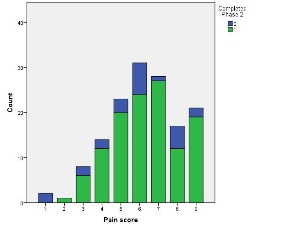

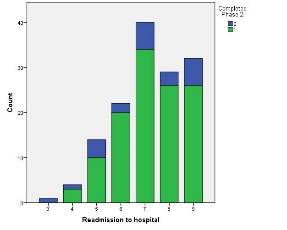

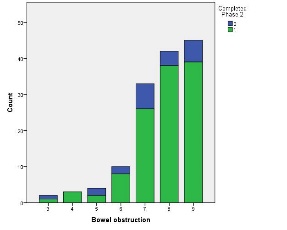

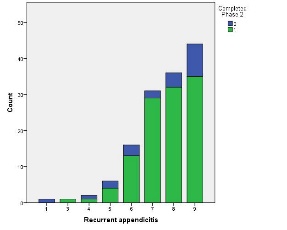

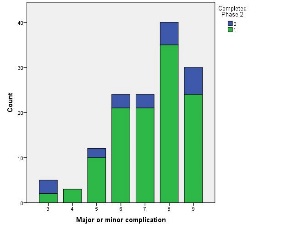

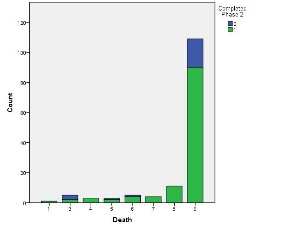

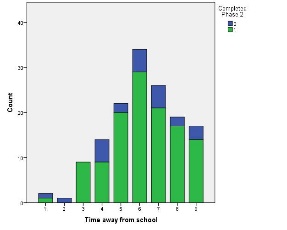

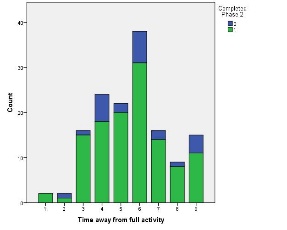

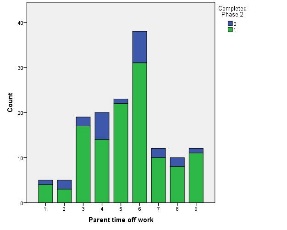

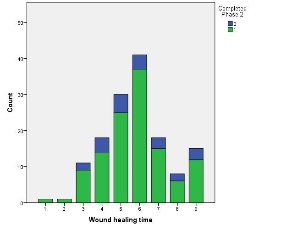

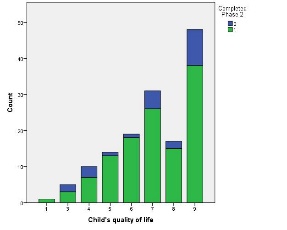

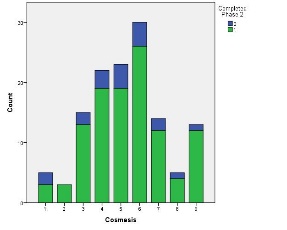

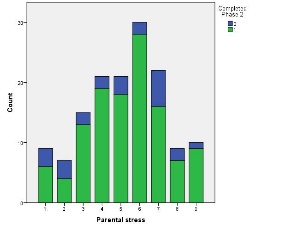

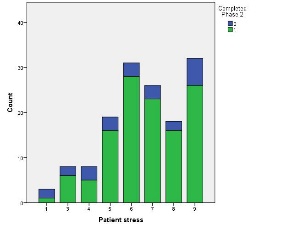

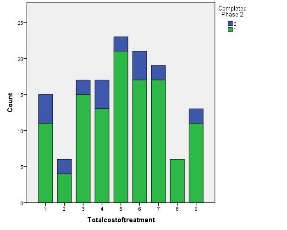

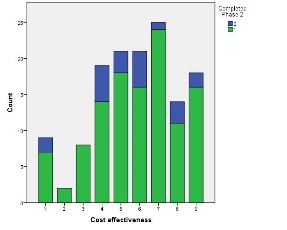

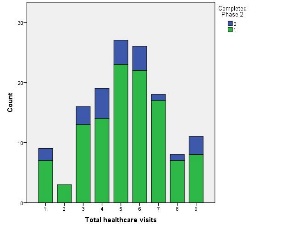

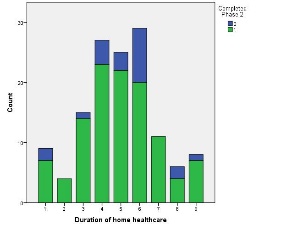


**Table S8** **Phase one scores compared between participants who completed phases one and three, and those who completed phase one only.**

| **Outcome** | **Phase 1 scores for completed phases 1 and 3,**  **Median (range)** | **Phase 1 scores for completed phase 1 only,**  **Median (range)** |
| --- | --- | --- |
| Operation time | 6 (2-9) | 6 (1-9) |
| Conversion to open operation | 6 (1-9) | 6 (1-9) |
| Blood Loss | 7 (1-9) | 7 (1-9) |
| Wound infection | 7 (1-9) | 7 (1-9) |
| Intra-abdominal abscess | 8 (3-9) | 8 (2-9) |
| Wound complication | 7 (3-9) | 7 (1-9) |
| Fever after treatment | 6 (1-9) | 7 (1-9) |
| Blood markers of inflammation | 5 (1-9) | 7 (1-9) |
| Other infectious complication | 6 (1-9) | 6 (1-9) |
| Duration of antibiotics | 6 (1-9) | 6 (1-9) |
| Recovery of bowel function | 6 (1-9) | 6 (1-9) |
| Time to ambulation | 6 (1-9) | 6 (3-9) |
| Hospital length of stay | 6 (1-9) | 7 (2-9) |
| Duration of drainage | 6 (1-9) | 6 (1-9) |
| Unplanned CT scan | 5.5 (1-9) | 5 (1-9) |
| Any unplanned imaging | 5 (1-9) | 5 (1-9) |
| Interventional radiology procedure | 6 (1-9) | 6 (1-9) |
| Unplanned central venous catheter | 6 (1-9) | 6 (1-9) |
| Re-operation | 8 (1-9) | 8 (1-9) |
| Antibiotic failure | 7 (1-9) | 7 (1-9) |
| Analgesia | 6 (2-9) | 6 (1-9) |
| Pain score | 6 (2-9) | 6 (1-9) |
| Readmission to hospital | 7 (4-9) | 7 (3-9) |
| Bowel obstruction | 8 (3-9) | 7 (3-9) |
| Recurrent appendicitis | 8 (3-9) | 8 (1-9) |
| Major or minor complication | 7.5 (3-9) | 8 (3-9) |
| Death | 9 (1-9) | 9 (3-9) |
| Time away from school | 6 (3-9) | 7 (1-9) |
| Time away from full activity | 6 (1-9) | 6 (1-9) |
| Parent time off work | 5 (1-9) | 6 (1-9) |
| Wound healing time | 6 (2-9) | 6 (1-9) |
| Child’s quality of life | 7 (3-9) | 7 (1-9) |
| Cosmesis | 5 (2-9) | 5 (1-9) |
| Parental stress | 6 (1-9) | 5 (1-9) |
| Patient stress | 7 (3-9) | 7 (1-9) |
| Total cost of treatment | 5 (1-9) | 5 (1-9) |
| Cost effectiveness | 6 (1-9) | 6 (1-9) |
| Total healthcare visits | 5 (1-9) | 5 (1-9) |
| Duration of home healthcare | 5 (1-9) | 5 (1-9) |
| Bacterial peritoneal cultures | 5 (1-9) | 5 (1-9) |

**Fig. S2 Phase one scores in participants who completed phases one and three (green), and those who completed phase one only (blue)**


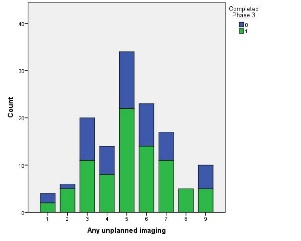

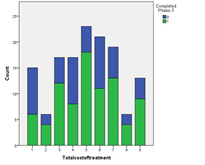

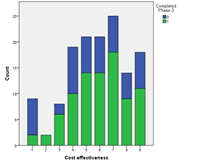

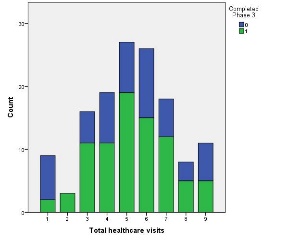

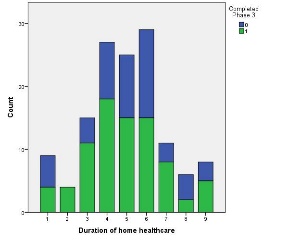

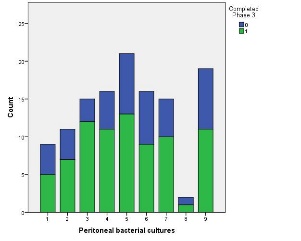

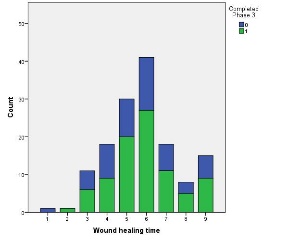

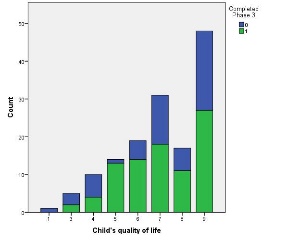

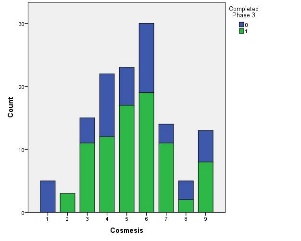

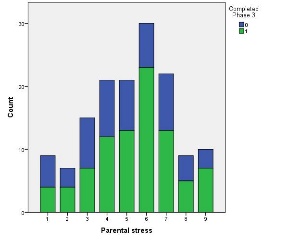

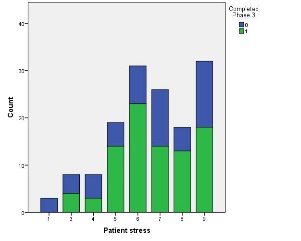

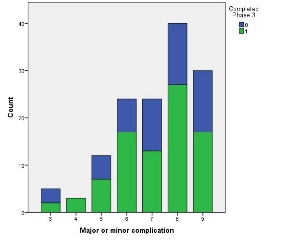

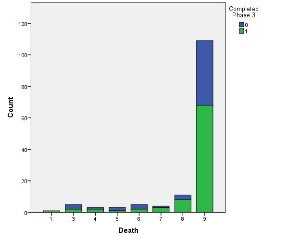

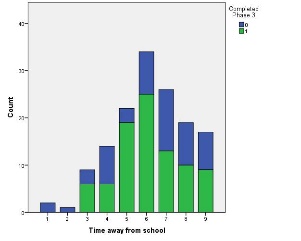

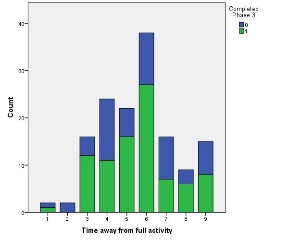

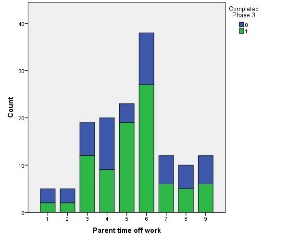

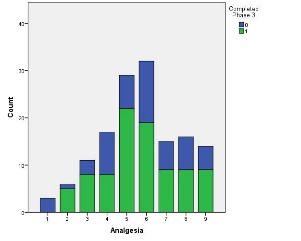

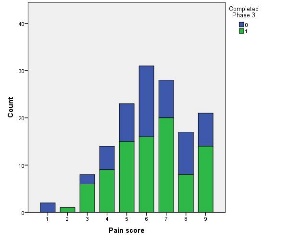

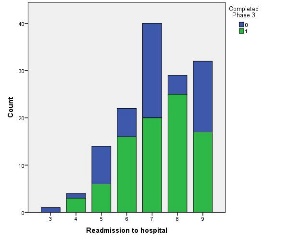

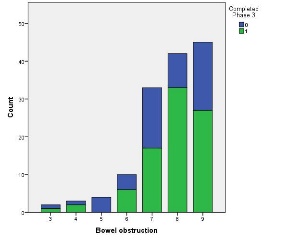

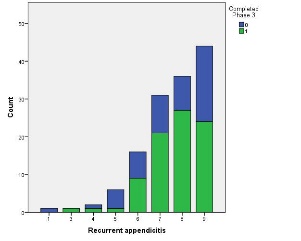

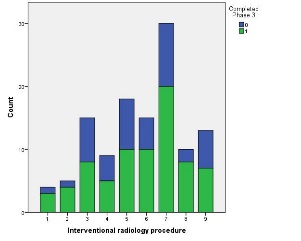

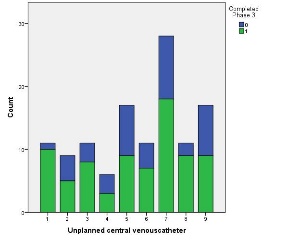

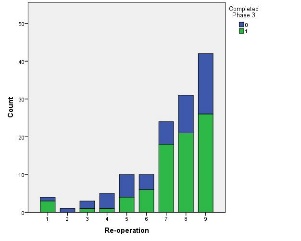

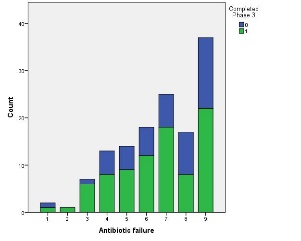

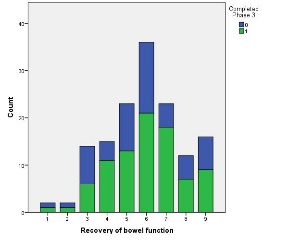

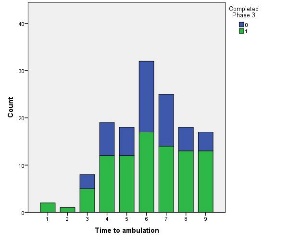

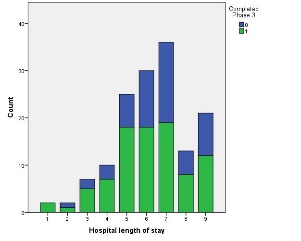

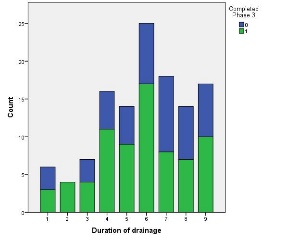

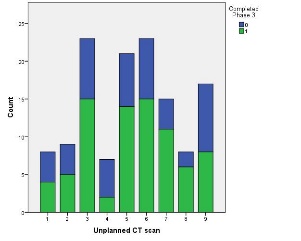

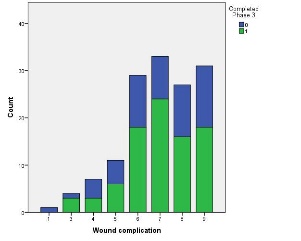

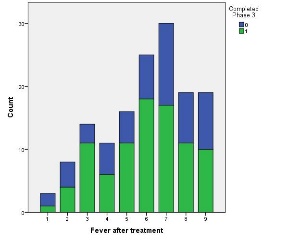

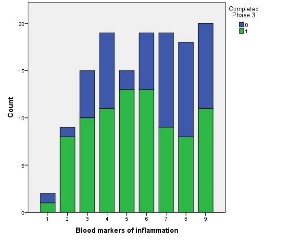

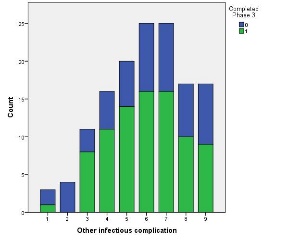

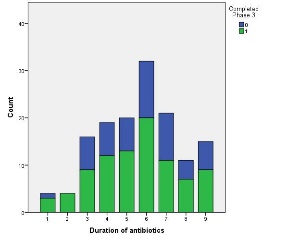

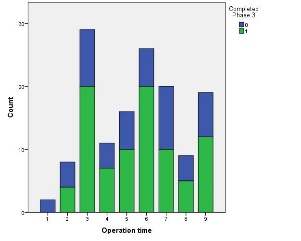

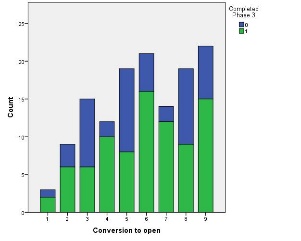

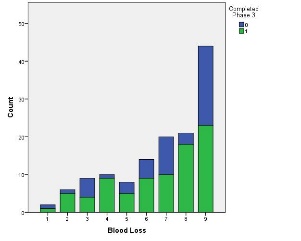

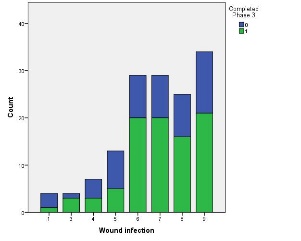

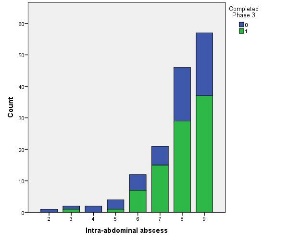

Supplement: Supplementary file 1 — Appendix S1. Supporting information [file BJS-107-1013-s001.docx]
